# Recombinant vaccines in 2022: a perspective from the cell factory

**DOI:** 10.1186/s12934-022-01929-8

**Published:** 2022-10-05

**Authors:** Marianna Teixeira de Pinho Favaro, Jan Atienza-Garriga, Carlos Martínez-Torró, Eloi Parladé, Esther Vázquez, José Luis Corchero, Neus Ferrer-Miralles, Antonio Villaverde

**Affiliations:** 1 Institut de Biotecnologia i de Biomedicina, Universitat Autònoma de Barcelona, Cerdanyola del Vallés, 08193 Barcelona, Spain; 2 Laboratory of Vaccine Development, Department of Microbiology, Institute of Biomedical Sciences, University of São Paulo, São Paulo, Brazil; 3 Centro de Investigación Biomédica en Red de Bioingeniería, Biomateriales y Nanomedicina, Instituto de Salud Carlos III, Cerdanyola del Vallès, 08193 Barcelona, Spain; 4 Departament de Genètica i de Microbiologia, Universitat Autònoma de Barcelona, Cerdanyola del Vallés, 08193 Barcelona, Spain

**Keywords:** Recombinant proteins, Vaccines, Antigens, Nanovaccines, Nanoparticles, VLPs

## Abstract

The last big outbreaks of Ebola fever in Africa, the thousands of avian influenza outbreaks across Europe, Asia, North America and Africa, the emergence of monkeypox virus in Europe and specially the COVID-19 pandemics have globally stressed the need for efficient, cost-effective vaccines against infectious diseases. Ideally, they should be based on transversal technologies of wide applicability. In this context, and pushed by the above-mentioned epidemiological needs, new and highly sophisticated DNA-or RNA-based vaccination strategies have been recently developed and applied at large-scale. Being very promising and effective, they still need to be assessed regarding the level of conferred long-term protection. Despite these fast-developing approaches, subunit vaccines, based on recombinant proteins obtained by conventional genetic engineering, still show a wide spectrum of interesting potentialities and an important margin for further development. In the 80’s, the first vaccination attempts with recombinant vaccines consisted in single structural proteins from viral pathogens, administered as soluble plain versions. In contrast, more complex formulations of recombinant antigens with particular geometries are progressively generated and explored in an attempt to mimic the multifaceted set of stimuli offered to the immune system by replicating pathogens. The diversity of recombinant antimicrobial vaccines and vaccine prototypes is revised here considering the cell factory types, through relevant examples of prototypes under development as well as already approved products.

## Introduction

Immune protection against infectious diseases is a main goal in human and animal health [1, 2]. The current vaccine narrative is flooded by COVID-19, for which an extremely rapid vaccination response has been imperative at global scale. Nowadays, vaccination has not only taken a dominant rule in the scientific literature but features of specific immunization strategies and vaccine-induced immune responses are also finely dissected and overtly discussed in the media. This situation has stressed the challenges posed by emerging viral pandemics and more generically, the transversal needs associated to vaccine development, irrespective of the involved pathogen-host pair [2, 3]. The usual failing in incorporating the whole infectious agent in a safe and protective vaccine formulation, either in inactivated or attenuated versions, pushes towards considering recombinant subunit vaccines [4, 5]. This is also supported by the inherent biological risks associated to bottlenecks in the large-scale chemical inactivation of pathogens, either bacterial cells or virus particles [6, 7], or to the potential of reverting to virulence in the case of attenuated strains [7–9]. Also, the manipulation of subunit vaccines can dismiss the use of P3 laboratories and high-biosafety facilities.

Being and old concept [10–12], subunit vaccines are based on particularly immunodominant antigens or cocktails of selected antigens purified from the pathogen. The main challenges in designing usable subunit vaccines are not only linked to their biological efficacy but also to the fastness, cost-effectiveness, biosafety issues and transversal nature of the process design and large-scale production. Therefore, the industrial-scale production of the relevant antigens is desired over their extraction from natural sources, often costly or even unfeasible. Importantly, the geometry of the selected antigen presentation might be critical [13–15], especially in anti-viral vaccines [16]. Then, virus-like particles (VLPs) have been achieved in different pathogenic and non-pathogenic viruses from more than 35 families through the spontaneous self-assembling of recombinant versions of capsid proteins, upon strong expression of the encoded genes [17]. VLPs have been developed as vaccines but also as carriers for drugs or imaging agents, proving the enormous potential of such technology in different fields of precision nanomedicines [18]. In immunization, VLP-based vaccines or vaccine prototypes have been generically successful probably because of the virus-like oligomeric presentation of the antigens [19]. However, so far, VLPs have been only approved for a limited number of diseases (Table 1). In a step further and following the concept of multiple and repetitive antigen presentation, vaccination platforms, namely versatile antigen presentation systems with modular or interchangeable elements are highly desired and pointed out as main goals for development [20, 21]. In the search of such transversal vaccine platforms, the multimeric antigen display on nanostructured materials has been repeatedly noted to favor, improve and enhance the protective response [22, 23]. Such nanoscale multimeric presentation mimics natural structural features of viral particles, what has prompted the development of universal antigen presentation systems in form of nanoparticles (nanovaccines) [24–27]. Because the specific and promising features of nanoscale vaccines, this strategy is given, as a global, a special attention in the next section.

**Table 1:** Main recombinant, subunit or oligomeric vaccines approved for human or veterinary use

	Pathogen	Antigen	Names	Year of 1st Approval (FDA/EMA)	Production system	Formulation
Human	Hepatitis B virus	Hepatitis B Surface antigen (HBsAg)	Engerix-B, Heplisav-B, Pediarix, HBVaxPRO, Recombivax, Twinrix, Vaxelis, Heplisav-B	1986	Yeast (*S. cerevisiae*) Yeast (*H. Polymorpha*)	VLPs
	Papillomavirus	L1 capsid protein	Gardasil, Gardasil 9 Cervarix	2006 2009	Yeast (S*. cerevisiae*) Insect cells-BVES	VLPs Adjuvanted
	Hepatitis E virus	ORF2 protein	Hecolin	2012*	*E. coli*	VLPs
	Influenza A & B virus	Hemagglutinin (HA)	Flublok, Flublok RIV4, Supemtek	2013	Insect cells-BVES	NPs No adjuvant
	Neisseria meningitidis serogrup B	2 fHbp variants	Trumenba	2014	*E. coli*	No adjuvant
	Malaria	HBsAg + RTS chimera	Mosquirix	2015	Yeast (*S. cerevisiae*)	VLPs
	Varicella Zoster virus	Truncated gE	Shingrix	2017	CHO cells	Adjuvanted
	SARS-CoV-2	Spike (S) protein	Nuvaxovid	2021	Insect cells-BVES	NPs
	SARS-CoV-2	Spike protein	Covifenz	2022**	Plant (*Nicothiana benthamiana*)	VLPs
Swine	A. pleuropneumoniae	ApxII, TbpB, CysL, Om1A proteins	Pleurostar APP	n.s	n.s	
	Classical Swine Fever virus	E2 protein	Porcilis Pesti, Bayovac CSF E2	2000 Withdrawn	Insect cells-BVES	Subunit Adjuvanted
	Porcine Circovirus Type 2	ORF-2 protein	Ingelvac CircoFLEX, Porcilis PCV, Circogard, Circumvent PCV	2008	Insect cells-BVES	Fom VLPs Adjuvanted
	Porcine parvovirus (PPV)	PPV 27a VP2	Reprocyc ParvoFLEX	2019	Insect cells-BVEs	VLPs Adjuvanted
Canine	Borrelia burgdorferi	OspA & C chimeric OspA	Vanguard crLyme, Recombitek Lyme	n.s	*E. coli*	No adjuvant
	Leishmania	A2 different species	Leish-Tec	2004	n.s	Adjuvanted
	Leishmania	Chimeric Protein Q	Letifend	2016	*E. coli*	No adjuvant
Poultry	Newcastle disease virus	Hemagglutinin-neuraminidase	Approved by USDA, not commercialized	2006	Plants (tobacco suspension cells)	
Feline	Feline leukemia virus	P45 env. antigen	Leucogen Nobivac LeuFel	2009	*E. coli*	Subunit Adjuvanted
Equine	*Streptococcus equi*	CCE, mEq84, IdeE	Strangvac	2021	*E. coli*	Adjuvanted

* Only approved in China; ** Only approved in Canada; VLP, virus-like particle; NP, nanoparticle; fHbp, lipidated factor H binding protein; gE, glycoprotein E; n.s., not specified; LiESP, L. infantum Excreted-Secreted protein, BVES, baculovirus expression system

This increase of the structural complexity in the formulation of subunit vaccines contrasts with the earlier single soluble antigen approach that initially arose linked to the recombinant DNA technologies at the late 70’s. Associated to vaccine formulation, stability during storage and transportation and the suitability of a vaccine product for mass administration need special attention [28]. Novel adjuvants and formulation strategies are being explored to allow the simplest manipulation of the vaccine doses [25, 29], especially at large scale administration programs in which thermal stability is particularly demanded [30].

In the context of emerging vaccination technologies, such as those based on expressible DNA or mRNA, recombinant antigens show remarkable interest. Recombinant proteins have been used as drugs for decades [31–34] and their intrinsic clinical safety and industrial scalability in their production have been largely demonstrated. In addition, the functional and structural versatility of polypeptides allow designing presentations as multimeric, nanoscale materials in which self-assembling is achieved through several alternative approaches [35–43]. This set of properties make them excellent candidates for new generation approaches in contemporary vaccinology. In addition, novel natural or engineered cell factories with appealing properties have been incorporated in the last decades to the biofabrication of protein drugs [44], beyond the more classical bacterial and yeast species and mammalian and insect cells. In the present review, we discuss the contemporary approaches in the biological fabrication of recombinant subunit vaccines from alternative cell factories and how these antigens are adapted to comply with the requirements for vaccine effectiveness regarding stability, formulation and multivalent nanoscale presentation.

## Nanovaccines

**Fig. 1:**
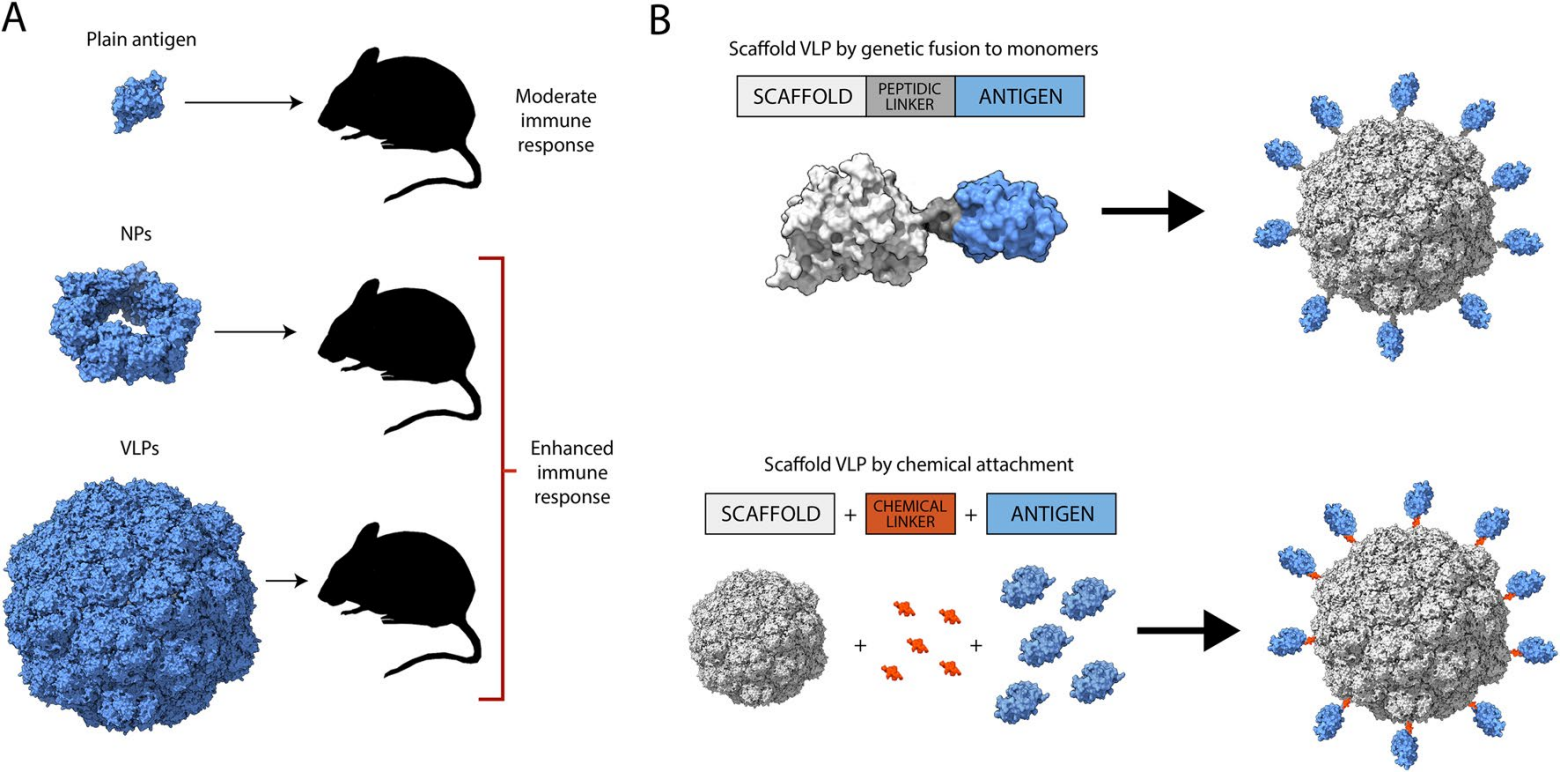
Principles of antigen engineering and formulation as nanovaccines. **A** The multimeric presentation of antigens, either as NPs or VLPs, enhances the protective immune response upon administration. **B** Non relevant NPs or VLPs can be used as scaffolds for the multiple presentation of antigens that are reluctant to oligomerization, either as genetic fusions of chemically coupled upon assembling of the scaffold protein

The immunogenicity of plain subunit vaccines, that is, non-oligomeric antigens, is often moderate. This fact is usually counteracted by combining proteins to immunostimulant molecules (adjuvants) and nanoparticles, which can also act as immune potentiators [45]. In the last decades, proteins have been displayed in multiple copies on a plethora of nanoparticle types, including metals, polymers, lipids, and others [13], aiming to increase half-life, promote a deposit effect, or to specifically activate certain immune cells [46]. However, the use of protein-only nanovaccines resulting from recombinant protein self-assembly has been also in use for a long time and represents a way to suit the requirements of an ideal vaccine formulation (Fig. 1). Also, it avoids the use of non-protein nanoparticles as carriers, what might represent an additional source of concern because of potential side toxicities in the case of xenobiotic, recalcitrant or poorly biocompatible materials [47–50].

The dedicated study of nano-sized oligomeric protein vaccines emerged with the development of VLPs, in which the multimerization of antigens seems to be essential for eliciting proper immune responses. The side-by-side presentation of antigens is recognized by immune cells as a pathogen-associated molecular pattern (PAMP) [13], which promotes the activation of B-cell receptors in a more efficient way since their cross-linking is a key and early step to activate B cells [51]. While the dense arrangement of antigens interacts with B-cell receptors, nanovaccines ranging from 20 to 200 nm in size are better drained to lymph nodes and thus allow a better uptake by antigen-presenting cells (APCs), which exemplifies how size and geometry of the protein assembly contribute to the immune responses [13]. The success of the VLP platform is evident by its presence in the market, with two formulations already being commercialized against Human Papilloma Virus (HPV) [52, 53] and two others against Hepatitis B Virus (HBV) [54, 55] (Table 1).

VLPs intended as immunogens for vaccination can be attained by producing the capsid of viral pathogens as recombinant proteins that thus self-assemble as an empty, viral-mimicking particle. In the case of proteins without the capacity to self-assemble, several tools can be used to modify scaffold, irrelevant VLPs to incorporate foreign proteins, such as genetic fusion of the antigen to coat proteins with the ability to self-assemble, or by conjugation of antigens to the scaffold through a variety of both covalent and non-covalent strategies [56]. A very promising and widely used strategy, named SpyTag/SpyCatcher, functionalizes a VLP expressing SpyCatcher sequence to covalently bind to recombinant antigens fused to a SpyTag sequence, thus allowing the decoration of VLPs by simply mixing them with the modified antigen [57]. This platform was evaluated against different pathogens and was recently applied to SARS-CoV-2, presenting encouraging results in pre-clinical trials [58]. In fact, the COVID-19 pandemics encouraged the expansion of different platforms of nanovaccines being assessed in pre-clinical trials. In this scenario, a recombinant nanoparticle vaccine based on the Spike protein from SARS-CoV-2 was developed by Novavax and it passed phase III clinical trials with 89.7% protection against infection [59], also showing cross-protection against antigenic variants [60]. Its approval represents an important step in the vaccinology against COVID-19 [61].

In light of all the potential advantages observed in the use of nanostructured subunit vaccines, a great deal of effort has been placed in the identification and development of alternative non-VLP strategies that allow self-assembly of antigens. Ferritin, for instance, is a popular platform which in different studies has been associated to an increased immune response towards the antigen, and lumazine synthase is also extensively used, and often preferred to recruit more subunits in the assembly than ferritin [62]. Overall, different protein oligomerization tags and strategies have been explored and developed [63], including the straightforward use of poly-histidine tails, common as purification tags, as self-assembling tools [40, 64, 65]. Despite the diversity of approaches, antigen assembly into a multimeric version consistently results in enhanced immune responses [66–70]. Altogether, nanovaccines represent an emerging field in the frontier between protein engineering and materials sciences. By combining the power and challenges of protein production in heterologous systems to the ones associated with nanoparticles, these tools provide an exciting opportunity to take subunit vaccines to the spotlight in terms of efficiency and safety.

## Approved recombinant vaccines

Many of the vaccines approved for clinical use still focus on using whole viruses or pathogen cells in conventional live or inactivated forms, being this fact particularly true for veterinary vaccines [71–73]. However, recombinant vaccines have gained interest as the application of recombinant DNA technologies in vaccinology solved most of the problems posed by the classic strategy, as discussed above. Therefore, the trend in immunization is shifting towards the exploration of this technology, as demonstrated by the significant number of approved recombinant vaccines (Table 1, [74, 75]). Among them, nanoparticle (NP) and VLP versions abound in this list, following the multimeric presentation principles discussed in the previous section. *Escherichia coli*, a few yeast species, insect cells, mammalian CHO cells and plant cells are the most common cell factories used for production, whose appealing properties and bottlenecks are highlighted and discussed in the next sections.

Most of these data are extracted from references [74, 75].

## Conventional cell factories for recombinant vaccines

### Bacteria

Bacteria are a straightforward choice to approach heterologous protein expression through recombinant DNA technologies. Their high productivity paired with the significantly lower culture cost of this platform when compared to other expression systems are very appealing features for large-scale protein production. Unfortunately, several issues inherent to the bacterial systems have hindered their adoption as preferred tools for recombinant vaccine production. These constraints revolve around the immunogenicity of residual bacterial components such as lipopolysaccharides (LPS) from the cell wall, or the inability to adequately fold complex proteins or those that undergo post-translational modifications when produced in their natural sources.

Regarding the first issue, procedures for LPS removal from protein preparations are under continuous development. In fact, these efforts have provided alternative methods for their generic and successful industrial application to any protein produced in bacteria and intended for clinical applications, including vaccinology [76–78]. In contrast, insolubility of recombinant proteins and the consequent precipitation as cytoplasmic or periplasmic inclusion bodies have represented a more persistent bottleneck in the biological fabrication of protein drugs in *E. coli* [79], reluctant to the generic application of most of the preventive metabolic strategies [80, 81]. This fact has limited the number of bacterial protein products transferred to the market, which are in fact those that naturally, or with cost-effective post-production protocols, reach a conformation compatible with solubility while retaining the expected biological activity. Alternatively, since inclusion bodies might contain high proportions of folded protein species [82, 83], strategies for the straightforward use of these protein aggregates as a formulation for recombinant antigens or immunomodulators are interesting and have offered intriguing data [84–87]. However, they have been in general discontinued. In addition, they face important regulatory issues because of their heterogeneous chemical composition [88].

*E. coli* stands as the most immediate host for recombinant protein production among suitable bacterial species due to its fast growth rate, a broad repertoire of genetic tools and a wide array of different strains engineered to solve most of the production challenges [89]. Accordingly, the three approved recombinant vaccines for human use that employ bacteria as the expression system are produced in *E. coli*. These vaccines are Hecolin (Xiamen Innovax Biotech, approved in 2012 in China), Bexsero (Novartis, commercialized since 2013) and Cecolin (Xiamen Innovan Biotech, approved in 2019), which offer protection against Hepatitis E, *Neisseria meningitidis* serogroup B and human papillomavirus 16 and 18, respectively (see Table 1). Outside of human application, there are other approved recombinant vaccines produced in *E. coli* prescribed to protect against canine, feline and equine pathogens, such as *Borrelia burgdoferi* (Vanguard crLyme, Recombitek Lyme), *Leishmania* (Letifend), Feline leukemia virus (Leucogen & Nobivac LeuFel) and *Streptococcus equi* (Strangvac) (see Table 1).

Although *E. coli* remains as the main expression system for recombinant production in bacteria, there are many other bacterial species being adapted in parallel that offer unique traits and advantages as protein producers. Among Gram negative bacteria, *Pseudomonas fluorescens* has been of particular interest for its non-acetogenic nature and moderate oxygen needs, enabling a lax control over oxygen and glucose concentrations while still achieving good production yields [90]. In fact, there is a wide toolbox and strains available for recombinant protein production that feature antibiotic-free selection or periplasmic protein expression [90–92]. To this date, two recombinant proteins produced in *P. fluorescens* have already been successful in eliciting protection against malaria and anthrax infections in mouse and rabbit models, respectively [93, 94]. Several other interesting gram-negative bacteria have been explored as potential options for expression systems, including *Pseudomonas putida*, *Ralstonia eutropha*, *Burkholderia glumae* and *Acinetobacter* sp., but broad host-range tools still need to be developed in terms of plasmid maintenance, plasmid transfer and secretion signaling [95].

Moreover, lactic acid bacteria and *Bifidobacterium* species are of particular interest due to their Generally Recognized as Safe (GRAS) status, as they do not require pre-market approval when used in the food industry. These bacteria have been massively employed by the industry and consequently, their engineering and scalability have been thoroughly explored. Based on the assumption of safety, the development of expression systems in several strains of such species has thrived as of lately. Many and diverse microbial antigens produced in or surface-displayed on lactic acid bacteria have been able to elicit significant immune and often protective responses [96–101]. These bacteria do not only serve as microbial cell factories [102, 103] but also as vaccine vectors [103, 104], for orally and mucosally-administered formulations, as they are able to induce a potent immune response by expressing foreign antigens, while drawing a minimal response against themselves [105–107].

All in all, the use of bacteria as the expression system for vaccine production is trending upward, with three approved vaccines in the last ten years. Moreover, the engineering and further tailoring of endotoxin-free *E. coli* strains might resolve one of the major issues of this expression system [108]. On the other hand, the currently ongoing efforts to export the *Campylobacter jejuni* N-glycosylation system to *E. coli* [109–111] could represent a breakthrough in the production of antigenic proteins for vaccination purposes in bacteria [110].

### Yeast

As it happens with other types of functional proteins, antigens intended for immunization might require natural post-translational modifications to follow a native folding and produce a 3D structure with proper epitope presentation, especially for those that are conformational rather than sequence-dependent [112–114]. Being good alternatives to bacteria and superior to them in solving some protein folding issues [115], yeasts are considered a useful system for the development of recombinant and unconventional vaccines for human and veterinary medicine [116–118].

Several yeast species are commonly used for recombinant protein production including *Kluyveromyces lactis, Schizosaccharomyces pombe, Arxula adeninivorans, Yarrowia lipolytica, Hansenula polymorpha, Komagataella phaffii* (also known as *Pichia pastoris*) and *Saccharomyces cerevisiae* [119]. From a metabolic point of view, these species can be categorized as methylotrophic and non-methylotrophic [120]. *S. cerevisiae*, the best-known member of the non-methylotrophic group, has provided important services in the bread and brewing industries for thousands of years. As a result of its nonpathogenic nature and lack of toxins, *S. cerevisiae* is one of the yeast expression systems most exploited to obtain biopharmaceutical products [31, 121–124]. It is also considered generally safe (GRAS) by the Food and Drug Administration (FDA) and an accepted food additive by the European Food Safety Authority (EFSA) [125]. However, major drawbacks include N-hyperglycosylation of proteins and low secretion efficiency. In fact, *S. cerevisiae* mannosylated glycoproteins are presumed to exhibit enhanced immunogenicity because they specifically interact with mannose-binding receptors found on professional antigen-presenting cells such as dendritic cells and macrophages [125].

In any case, genes involved in the hypermannosylation process have been identified in *P. pastoris*, and mutations in these genes can reduce the production of undesirable glycoforms [126]. Therefore, glycoproteins derived from *P. pastoris* can act as adjuvants. *P. Pastoris* is a methylotrophic yeast that has proven to be an excellent host system for recombinant expression. In addition to being inexpensive, this yeast system is fast in terms of expression times, as well as in co-translational and posttranscriptional processing. The cultivation of this species in industrial bioreactors allows the production of large amounts of recombinant protein from high-density cell cultures [126].

Various viral proteins from hepatitis viruses [127], papillomavirus [128], porcine circovirus [129] or influenza virus [130], among many others, have been produced in *P. pastoris*, either in the form of viruses like particles, purified protein, surface display or as a whole recombinant yeast cell [118]. Different candidate vaccines based on antigens, toxins, or VLPs are currently under development. Representative prototype examples are a subunit RBD vaccine against COVID-19 [131, 132], a CRM137 subunit vaccine against Typhoid fever caused by *Salmonella enterica* [133], a LipL32 subunit vaccine against leptosipirosis caused by *Leptospira interrogans* [134], a NS1-based VLP vaccine against Zika virus disease [135] and an HEV-VLP against hepatitis E [136].

Strain engineering (e.g., glycosylation) and process engineering (e.g., continuous processing, alternative induction systems) will not only result in a greatly improved productivity of the *P. pastoris* system, but it is also expected that these changes will expand the range of available immunogens, including those currently only accessible through other expression methods. This improvement strategy considers the endpoint of application before deciding how heterologous proteins are produced. Therefore, we can expect *P. pastoris* to become one of the first platforms of choice in the coming years as the biopharmaceutical application of this host system improves.

### Insect cells

The insect cell-baculovirus expression system (BVES) is a robust platform that supports the production of different protein formats [137]. The ability of insect cell lines to grow in suspension in serum-free medium and to reach high density conditions positions this system as one with interesting advantages for the production of immunization agents [138]. In addition, this system allows the introduction of several genes in the baculovirus genome backbone under control of the strong viral promoters [139] and the use of recombinant baculovirus as transduction vectors for mammalian cells in the BacMam expression system [140]. However, even though insect cells BVES reproduces most of the post translational modifications of mammalian cells, the distinctive glycosylation pattern results in the synthesis of asialylated glycoproteins [141]. Another drawback when comparing with mammalian cell-based expression systems is the need to control and maintain the viral stock since the expression experiments are based on a batch procedure, and each infection regime implies the control of the cell culture but also the quantification of the viral suspension which is time consuming and expensive. Although this platform is considered more biosafe than the expression system based on mammalian cells, infectious agents with potential risk for human and animal health have been detected and need to be screened [142].

As in the mammalian expression system, the protein product can be accumulated intracellularly or sent to the medium by the addition of optimized insect secretion signals in the recombinant gene [143]. However, more sophisticated protein presentations are obtained such as VLPs. These multiprotein structures can be isolated from the soluble cell fraction or from the medium when the membranous VLPs are budded [144–146].

The BEVS is well positioned in the vaccine area for veterinary applications (Table 1). In fact, several products from this expression system have been approved by the EMA and FDA to fight against main viral pathogens of swine (*African swine fever virus*/ASFV, *Porcine circovirus 2*/PCV-2 and *Ungulate protoparvovirus 1*/PPV) [147]. The composition of the vaccine products for ASFV is based on subunits of glycoprotein E2 of the outer envelope of the virus (Procilis® Pesti and Bayovac CSF E2®) [148] while the ones for PCV-2 and PPV are VLPs of capsid protein VP2 for PCV-2 (Ingelvac CircoFlex®, Porcilis® PCV, Circogard®, and Circumvent® PCV) [149–151], and the corresponding VP2 for PPV (Reprocyc® ParvoFLEX) [152]. The initial approvals of recombinant vaccines for veterinary use were based on subunits, moving to multiprotein complexes as VLPs. In fact, the vaccines based on protein subunits for circovirus have been withdrawn and only higher order protein complexes are keeping the authorization status after review (Table 1).

In the case of human vaccines, formulations based on protein nanoparticles (NPs) and VLPs have been recently approved. Cervarix® is a vaccine against two viruses of the *Papillomaviridae* family which are related to cancer and is formulated as VLPs formed by the spontaneous assembly of the L1 capsid protein of the corresponding viruses [153]. In the production of the bivalent vaccine, two independent infection processes are performed, each with a recombinant baculovirus with the corresponding cloned L1 gene. The VLPs are independently purified and mixed for the final formulation of the vaccine. This type of vaccine has also been obtained from yeast (Gardasil®) to include VLPs from type 16 and type 18 human papillomaviruses, and is even formulated as a quadrivalent vaccine when VLPs of type 6 and type 11 human papillaviruses are added, after their production in separate fermentations [154]. Even when data on the pathogenicity of other papillomaviruses has become available, Cervarix® has not been reformulated to increase the number of distinct VLPs. Menwhile, a new nonavalent formulation of Gardasil® was approved including five more distinct virus VLPs in the Gardasil® 9 formulation [155]. In 2013, an alternative to egg-based vaccine for seasonal influenza infections, Flublok®, was approved. In this case, due to the antigenic drift of the seasonal flu viruses, it is necessary to review vaccine formulation every epidemic season for a trivalent vaccine of two seasonal virus of *Influenza A virus* and one seasonal virus of *Influenza B virus* [156]. In 2016, a quadrivalent version of Flublok® was approved to include VLPs of the haemagglutinin of a second seasonal virus of *Influenza B virus* to the trivalent formulation. Finally, a recombinant vaccine based on the spike protein (S) of SARS-CoV-2 (Nuvaxovid®) has been approved [157]. In this instance, the full-length S glycoprotein self-assembles into trimeric complexes which organize into higher-order structures at the nanoscale.

Several strategies are being developed to improve the insect cells BVES including the humanization of the glycosylations pattern [158], the delay of the apoptosis process in infected cells [159], the optimization of the secretion pathway and the control of proteolysis [160] among others. Another interesting strategy that brings this expression system, close to the one based in mammalian cells, is the establishment of transient gene expression procedures or stable transgenic cell lines with the use of strong insect cell promoters [161, 162].

Therefore, the insect cell-BVES, is a flexible expression system platform which supports the production of complex heterologous proteins. The synthesized proteins adopt conformations compatible with the formation of higher-order complexes with potential for nanovaccine development. Further improvements to overcome the limitations of this expression system are being explored as of now, which could help to secure its positioning in the vaccine market.

### Mammalian cells

Before the development of cell culture technologies, the few available viral vaccines, based on whole virus particles, were produced in animal systems such as calf skin (smallpox), rabbit spinal cord (rabies), mouse brain (Japanese encephalitis), or embryonated eggs (influenza and yellow fever viruses) [163]. Currently, embryonated eggs are still a main source of conventional, whole virus vaccines, especially for the seasonal flu [164]. However, the use of eggs for vaccine production poses a number of concerns regarding the risks of insufficient supply especially in case of epidemics and pandemics, time-consuming procedures, increased manufacturing costs and the potential allergic responses to eggs components [165]. Cell culture technology appeared as an approach to overcome limitations of egg-based vaccine production and it was progressively incorporated. The development of the polio vaccine in 1954 by Jonas Salk is considered a milestone in the field of vaccination. Later, a vaccine for rubella was obtained from cultured human cells by Stanley Plotkin at the Wistar Institute (Philadelphia). There, Plotkin developed a cell line (named WI-38) from lung cells of an aborted fetus, in which many viruses, including rubella virus, could be grown. Since their establishment in the 1960s, cell lines WI-38 and MRC-5 (also initiated from fetal lung cells) have been used to produce several viral vaccines based on infection, whole virus recovery and further attenuation or inactivation, like those for hepatitis A (VAQTA, Merck and Havrix/GlaxoSmithKline), rubella (MERUVAX II, Merck, and ProQuad/Merck), chickenpox (Varivax, Merck and ProQuad/Merck), shingles (zoster) (Zostavax, Merck), oral vaccine against adenovirus type 4 and type 7 (Barr Labs), and rabies vaccine (IMOVAX, Sanofi Pasteur). Compared to egg-based vaccine production, mammalian cell cultures provide shorter production times in more controlled processes that takes advantage of closed-system bioreactors, and the opportunity to cultivate viral stocks without significant egg passage-dependent antigenic changes [166].

Apart from its role in whole virus production, immortalized mammalian cell lines are efficient factories for recombinant protein production, also with the ability to make complex and precise post-translational modifications, essential for correct folding and most likely needed to mimic the antigenic structural and glycosylation patterns the host encounters during natural infection [167]. However, the possibility for cell lines to carry mammalian pathogens or their potential tumorigenicity are considered key disadvantages for mammalian cells as producers of therapeutic molecules [168, 169]. In this regard, the Vero cell line was established in the early 1960s from kidney cells of an African green monkey in 1962, and was the first cell line approved by the WHO to produce viral vaccines for human use under specified regulatory guidelines [170]. Vero cells are considered non-tumorigenic below a certain passage number and safe to use as a substrate for vaccines, including vaccines against Japanese encephalitis, rotavirus, polio, influenza or smallpox [170, 171]. More recently, in a recent study to develop a candidate VLP vaccine for COVID-19, a stable SARS-CoV-2 VLP has been produced using the Vero E6 cell line [172].

Apart from Vero cells, other cell lines such as Chinese hamster ovary (CHO), baby hamster kidney (BHK), human embryo kidney (HEK), CAP‐T cell line derived from human amniocytes, and east lansing line-0 (ELL-0) are extensively utilized for the production of recombinant VLPs. CHO cells, the most frequently used cell line, have an additional advantage over other cell lines due to its non-human origin, which prevents the risk of contamination with human pathogens [146]. Additionally, CHO-based systems can be considered safer and cheaper than, for example, those based on recombinant lentivirus, which require a higher biosafety capacity [173, 174]. CHO cells growing in suspension in serum-free medium can be used to produce recombinant viral proteins, such as the S and PreS2 proteins of the hepatitis B virus (HBV) surface antigen, which are then assembled into HBV-like particles [175]. Indeed, the GenHevac® B vaccine, which contains these viral proteins, is immunogenic in humans [176]. The cytomegalovirus (CMV) glycoprotein B antigen has also been stably expressed in CHO cells, leading to the development of a recombinant vaccine that is immunogenic in humans [177, 178]. Recently, CHO cell line was used by Glaxo Smith Kline (formerly Novartis Vaccines) to produce a pentameric molecule consisting of the human CMV surface proteins. The pentamer could be recognized by monoclonal antibodies, and induced neutralizing antibodies in mice, suggesting its suitability as a vaccine in humans [179].

The third generation of the hepatitis B vaccine, Sci-B-Vac, contains three HBV antigens, including the S, Pre-S1, and Pre-S2 antigens, and is also expressed in mammalian CHO cells [180]. CHO cells have also been used to produce Hantavirus VLPs, which increase CD8^+^ T cell activity and induce antibody responses comparable to those seen with inactivated vaccines [181]. There is a human vaccine approved for human use (see Table 1) that is produced in CHO cells. Shingrix (GSK, Londres, UK) is a herpes zoster vaccine based on varicella zoster virus glycoprotein E [182].

Another widely used mammalian cell line, the HEK293 cell line, was created by transfection of a human primary embryonic kidney cell culture taken from an aborted embryo with sheared DNA of adenovirus type 5 (AD5) [183]. The advantages of HEK293 cells are their ability to grow in suspension in serum-free medium, suitability for large scale transient gene expression, high transfectability and stable expression. Two genetic variants have been described for the HEK293 cell line: the 293E line and the 293 T line, expressing the EBNA-1 antigen and the Simian Virus 40 large T Ag, respectively. These cell lines sustain episomal replication of plasmids containing the EBV and SV40 origins, respectively [184]. As with the EBNA1-expressing CHO cell line, the fact that these HEK293 genetic variants constitutively express viral antigens could present challenges for health authority approval. In addition, the tumorigenicity of this cell line is still an issue [185]. An Ebola virus (EBOV) VLP vaccine candidate has been produced by expressing the EBOV VP40 and the virus envelop glycoprotein in HEK 293 T cells. These VLPs were morphologically similar to wild-type virus particles, highly immunogenic in in vitro and in vivo studies, and they effectively induced the maturation, activation, and secretion of cytokines and chemokines. Mice vaccinated with EBOV VLPs showed B cell activation and produced high levels of EBOV-specific antibodies. The VLPs also activated CD4^+^ and CD8^+^ T cells and protected mice from deadly challenges [186]. Nipah virus VLPs can also be formed in HEK293T cells expressing the virus attachment glycoprotein (G), fusion (F) glycoprotein and matrix (M) protein [187]. Mice vaccinated with such VLPs produce specific antibodies against Nipah virus and a strong CD8 + T cell response. Neutralizing antibodies have also been observed in pigs vaccinated with NiP VLPs, but in these animals no CD8 + T cell responses were detected [188]. VLPs generated using proteins from other paramyxoviruses have also been developed, and have shown promising results in initial pre-clinical studies [189]. The COVID-19 pandemic drove the rapid development of adenoviral vectored vaccines and their eventual emergency use. Among the adenoviral vectored vaccines approved for emergency use by the WHO, Ad5-nCOV [190] and ChAdOX1-nCoV [191] are produced in HEK293 cells, while Ad26.COV2-S is produced in PER. C6 cells [192].

Regarding the expression system itself, recombinant proteins can be expressed transiently or stably. Mammalian cells constitutively producing recombinant proteins are established by inserting the recombinant gene into the host genome, a costly and time-consuming process. Even though stable cell lines based on CHO cells are widely used to produce recombinant proteins [193], there are inherent limitations in the synthesis and secretion of many complex polypeptides, such as low productivity, growth restriction and expression instability, low resistance to culture-related stresses and high costs of production. Random insertion of the foreign gene into the host genome can result in clonal genotypic variation and phenotypic instability (which jeopardizes cell line stability and process reproducibility and consistency), and in genomic instability over time, causing a drop in protein production [194, 195]. All these facts complicate the procedure and increase the production costs [196]. Thus, improvement of stable cell lines has become a clear need, using strategies involving genetic modification, optimization of expression vector and process engineering [197, 198]. With the advancement of CHO cell line development and process optimization, yields of some recombinant proteins (such as monoclonal antibodies) have achieved as high as 5 g/L, or even more than 10 g/L [199, 200]. Faster and cheaper approaches for protein production are preferred when many proteins (or several variants of a single protein) must be rapidly obtained and evaluated. In this context, transient gene expression (TGE) is the strategy of choice. TGE has a relatively short period for protein harvesting, but usually results in low yields, as the foreign gene is not integrated into the host genome and therefore is lost through time [201]. The TGE efficiency using HEK cells (the leading human cell line platform used in this approach) is restricted by low transgene expression levels [202]. Therefore, transient expression systems are only of short-term usage [203]. Besides, proteins transiently expressed can show heterogeneity in glycan content, resulting in inconsistency in affinity and efficacy [204]. Finally, another general drawback of human cell lines (like HEK) is that they are vulnerable to human viral infections. Thus, viral inactivation on human cell lines is essential [205].

In conclusion, despite these limitations associated to low yield anf high cost, mammalian cell cultures provide a flexible and scalable platform that can benefit from well-established biopharmaceutical bioreactor cell culture infrastructures for vaccine production. The combination of advances in cell culture as the use of serum-free medium, suspension culture, microcarriers to increase cell densities and improvements in bioreactor design result in a greatly improved strategy to produce new and more effective vaccines for human and animal health.

## Alternative factories for recombinant vaccine production

Despite the success of the aforementioned systems to produce recombinant proteins, other approaches are also available, probably being plants the most relevant alternative platform as vaccine producers. The production of therapeutic proteins in plants is often referred to as molecular farming, a method first proposed in 1986 by introducing a human growth hormone gene in tobacco and sunflower plants [206]. Since then, different plants were modified to express recombinant proteins including but not limited to maize, tobacco, potato and rice. After some therapeutic proteins were successfully produced, the first plant-produced vaccine was approved by the US department of agriculture for veterinary use in 2006. It consists of a subunit vaccine against Newcastle disease virus (NDV) produced in cultured tobacco cells that successfully protected poultry from a challenge with NDV [207, 208].

A hallmark for plant-made vaccines was the production of 10 million doses of a candidate H1N1 influenza virus vaccine composed of a plant-produced VLP within a month of receiving the sequence information, presenting a serious advantage in relation to the traditional egg-derived influenza vaccines that are limited by time-consuming and hard-to-scale manufacture [209]. The plant-produced VLPs were successful in preclinical trials and were able to induce both humoral and cellular immune responses in phases I and II trial in humans, presenting a satisfactory safety profile and modest results in phase III trials [210–212]. In the last years, several plant-based vaccine strategies are being assessed in clinical trials, developed against hepatitis B, cholera, Ebola, influenza, and other infectious diseases [213]. More recently, the COVID-19 pandemics pushed plant-made vaccines to the spotlight, with 3 phase 3 trials taking place targeting SARS-CoV-2 and presenting encouraging results [214]. Among them, Medicago® employed the same VLP technology initially developed for influenza and managed not only to reach a high rate of protection against different SARS-CoV-2 strains but also to become the first plant-produced vaccine approved for human use [215, 216].

Even though plants can be stably modified, most approaches focus on transient expression, frequently using *Agrobacterium tumefaciens* or to a lesser extent other viruses such as Tobacco mosaic virus (TMV), a process that is less time-consuming and renders higher and more consistent yields [213]. Lately, chloroplast transformation has emerged as an alternative since it allows proteins to fold and accumulate in the subcellular compartment, even though this strategy still faces drawbacks regarding low efficiency and impaired glycosylation [217]. In fact, glycosylation of plant-produced proteins is a relevant aspect of this system since while this process does occur, plant glycans differ slightly from human ones. This may render an increased immunogenicity. While this feature may be deleterious for therapeutic proteins for reducing their efficacy and likely eliciting side effects, it may actually boost the effect of subunit vaccines. The configuration of plant polysaccharides may act as a natural adjuvant, as they bind to receptors expressed by APCs and are recognized as PAMPs, which enhances antigen presentation by APCs [218, 219]. In fact, several plant polysaccharides are currently being explored as adjuvants, with encouraging results that allowed many of them to advance to clinical trials [218]. In response to concerns about allergies and immune responses elicited against the glycosylation pattern, several strategies have been developed to modify plant glycosylation patterns to be more human-like, mainly focusing on modifications of N-glycosilation or syalation pathways [209, 217]. Glycoengineering is evolving up to a point where even entire human glycosylation biosynthetic pathways can be transferred into plants, combined to the elimination of unwanted native glycosylation enzymes, aiming to increase product quality and safety [217, 220].

Overall, plant-produced vaccines present several advantages, as the production in this system tends to be cheaper than in mammalian cell cultures but it allows relatively similar protein folding, assembly and glycosylation, and unlike bacterial systems it renders endotoxin-free products [221, 222]. Scale-up of production is easier as it consists of simply growing more plants rather than relying on bioreactor production optimizations, and plant-based vaccines frequently dismiss the use of cold-chain for transport [222]. This system requires less sophisticated infrastructure than its counterparts and could help overcome vaccine distribution issues around the globe. On the other hand, some concerns have been raised regarding the use of genetically modified plants. Environmental risks such as gene transfer or exposure to proteins used as selectable markers, as well as inadvertent exposure to the engineered antigens, are factors to be considered to establish the proper crop management [222]. Concerns related to pollen improper dispersion are being addressed by using tissue-specific promoters that drive the transgene expression to specific tissues. Beyond this, the downstream processing for protein purification can become expensive as copious volumes of impurities and cell debris must be removed, which reduces the financial advantage obtained by a cheaper production [219]. Overall, plant-based vaccines can particularly thrive whenever an orally-administered formulation is viable, such as for veterinary use, or when the speed of production is an important feature, such as for emerging diseases, since transient expression is easier to scale-up in this system [219].

Another alternative system is the use of algae to produce recombinant proteins, and while different species can be used, the most advanced microalgal platform is *Chlamydomonas reinhardtii*, which has already been used to produce several proteins. In this case, the gene insertion is often performed in the chloroplast, which further allows the accumulation of the protein of interest and thus facilitates the downstream purification steps [223]. In 2003 the first algae-produced vaccine was reported [224]. It was followed by reports of antigen-specific antibodies being elicited against the structural protein E2 of classical swine fever virus (CSFV) after mice were vaccinated with antigens produced in *C. reinhardtii* [225]. Alternatively, these algae can be used for the administration of edible vaccines for farmed animals such as fish and poultry, being dried and formulated along with the feed. For instance, a malaria vaccine composed of freeze-dried algae that produced a *Plasmodium falciparum* surface protein was used to orally immunize mice and was able to elicit both IgG and IgA immune responses [226]. Despite its potential, it is noteworthy that this system lacks the post-translational modifications.

Transgenic animals have also been investigated as an alternative for recombinant protein production, mainly due to the possibility of producing large amounts of complex proteins in the milk [227]. In the context of vaccine production, two rotavirus proteins were produced in the milk of transgenic rabbits. The yields were up to 200 μg/ml and they were able to induce antigen-specific immune responses that conferred a high level of protection against virus challenge, either when partially purified milk was intrarectally administered or when whole-milk was orally administered to mice [228, 229]. In another study, mice were modified to produce an enterovirus 71 protein in the milk, and pups receiving transgenic milk orally developed antigen-specific antibodies and demonstrated relatively better health conditions after the challenge [230]. However, in the last years, there are less studies published that use transgenic animals as expression systems.

## Recombinant vaccines for COVID-19

Beyond the approved anti-COVID-19 front-line vaccines based on expressible embedded mRNA and replication-incompetent adenovirus vectors [118, 231, 232], a recombinant vaccine has come to market (Table 1, [157]) and other many alternative recombinant vaccines are also approached through under fast developing strategies. Because of the broad clinical experience in protein-based vaccination, such protein-based vaccines are expected to enter the prophylaxis landscape and complement or even substitute, in the longer term, the nucleic acid versions [233–236]. Due to the enormous investment in testing immunization strategies against SARS-CoV-2 infection, a comprehensive and updated overview of the spectrum of vaccine prototypes is just not feasible and it is beyond the scope of this review. However, it can be stressed that most of the taken approaches are based on the full-length spike protein (S) or its receptor binding domain (RBD), responsible for virus attachment to the host cell. Other SARS-CoV-2 proteins are also under consideration as epitope-full, including E, N and M [237, 238], with are explored in essentially all the protein production systems discussed above.

## Conclusions

Through the recruitment of technologies and expertise from more than 40 years of recombinant drug production [31, 34], vaccinology has moved from the conventional use of attenuated or inactivated live vaccines to recombinant antigens in different formulations. In particular, the presentation of vaccine candidates in form of oligomeric NPs or VLPs, mainly pushed by the development of insect-based protein production platforms, has allowed to overcome the moderate immunogenicity of plain subunit vaccines. The multiple and regular antigen presentation at the nanoscale boosts the immune response and increases protection, as well as proper antigen folding promoted by a native-like glycosylation pattern. Oligomerization is achieved not only in insect cell platforms but also in other production systems. The convenient formulation of the vaccine candidates is largely favored by the extension of the catalog of production platform explored for recombinant vaccine production, which include transgenic plants and animals. Despite the enormous success just observed by the application of mRNA technologies in vaccination against COVID-19, recombinant vaccine formulations still have plenty of room for engineering and improvement, and they show potential to overcome some of the limitations posed by genetic vaccines.

## Data Availability

Not applicable.
